# Permanent first molar eruption failure in children: leading signs for early diagnosis

**DOI:** 10.1186/s40510-025-00570-6

**Published:** 2025-07-03

**Authors:** Cristina Grippaudo, Elisabetta Tabolacci, Marco Farronato, Pietro Chiurazzi, Sylvia A. Frazier-Bowers

**Affiliations:** 1 Dipartimento Universitario testa collo ed organi di senso, Università Cattolica del Sacro Cuore, 00168 Rome, Italy; 2 UOC Clinica Odontoiatrica, Dipartimento di Neuroscienze, Organi di senso e Torace, Fondazione Policlinico universitario A. Gemelli, IRCCS, 00168 Rome, Italy; 3 Dipartimento di Scienze della Vita e Sanità Pubblica, Sezione di Medicina Genomica, Università Cattolica del Sacro Cuore, 00168 Rome, Italy; 4 Fondazione Policlinico Universitario A. Gemelli IRCCS, 00168 Rome, Italy; 5 Department of Biomedical Surgical and Dental Sciences, University of Milan, 20122 Milan, Italy; 6 Fondazione IRCCS Ca’Granda Ospedale Maggiore Policlinico, Milan, Italy; 7 UOC Genetica Medica, Fondazione Policlinico Universitario “A. Gemelli” IRCCS, 00168 Rome, Italy; 8 Richard and Nancy Christiansen Distinguished Professor, Orthodontics and Oral Facial Genetics Dean, Indiana University School of Dentistry Indianapolis, Indianapolis, IN 46202 USA

**Keywords:** Permanent molar eruption failure, Primary failure of eruption (PFE), Ankylosis, Mechanical failure of eruption (MFE), *PTH1R* gene, Infraocclusion, Pediatric dental anomalies, Eruption failure diagnosis, Genetic testing in dentistry

## Abstract

**Background:**

This cross-sectional observational study seeks to determine the clinical differences in eruption failure of permanent first molars presenting in cases of ankylosis, failure due to mechanical obstruction (MFE), and failure due to genetic causes (PFE). A total of 34 patients between 7 and 12 years old (mean ± SD: 9.3 ± 1.28 years), with anomalies in the eruption of the first permanent molars, were selected based on clinical observation, the evaluation of orthopanoramic radiographs, and intra- and extra-oral photographs. Genetic testing was also conducted to identify variants of the *PTH1R* gene in 27 patients with clinical signs of PFE. The familial nature of the condition was investigated through anamnesis of the first-degree relatives.

**Results:**

Out of the 34 patients, 3 were diagnosed with PFE, confirmed by the presence of *PTH1R* variants. Twelve patients showed clinical signs suggestive of MFE diagnosis. The remaining 19 cases, in which no variants of the *PTH1R* gene were found, were considered cases of ankylosis. Roots in ankylosed teeth were located in the basal bone and often dilacerated. The reduction of vertical growth of the alveolar bone was present in both PFE and ankylosis cases, but teeth were nearer to the basal bone in ankylosis cases. Infraocclusion of deciduous teeth was present in PFE and MFE cases. Asymmetry due to bilaterally unbalanced eruption of the teeth was present in six cases with ankylosis. Bilateral affection was noticed in one PFE case and 6 MFE cases. A descriptive statistical analysis using Fisher’s exact test was employed to evaluate the significant association between variables.

**Conclusions:**

The study highlighted some characteristic signs that help in early diagnosis of cases of PFE, MFE, and ankylosis. However, genetic testing remains necessary to understand the nature of the most dubious cases.

## Background

Tooth eruption is characterized by movement of the developing tooth from its origin in the bone to the functional position in the oral cavity [1]. In the mouse model the eruption process is orchestrated by stem cells surrounding the tooth and the Periodontal Ligament (PDL). Development of the PDL is determined by the *Pth1r* expression, a protein-coding gene that encodes the parathyroid hormone receptor [2, 3]. During this process, an eruption failure can occur, which could be diagnosed as Primary Failure of Eruption (PFE; OMIM # 125350) or ankylosis. In the case of PFE, the loss of function of Pth1r within Pthrp-expressing cells affects PDL and root phenotype, determining the failure of eruption [4]. This condition is more evident in the first permanent molars, and the eruption phenotype is partially penetrant. The eruption pathway is clear because the parathyroid hormone-related protein (PTHLH; OMIM168470) in dental epithelial cells in the coronal portion is expressed normally, while it is insufficient in the mesenchymal cells surrounding the roots. In humans with pathogenic variants in *PTH1R* (Parathyroid Hormone 1 Receptor, OMIM168468), the eruption pathway is clear, but the eruption stops prematurely, and the affected tooth partially erupts. Orthodontic forces can be ineffective in moving the affected teeth. *PTH1R* is a receptor for both parathyroid hormone (*PTH*; OMIM 168450) and parathyroid hormone-related protein (*PTHLH*). Earlier studies described the involvement of the *PTH1R* gene in 2010 [5, 6]. Previously published studies generally refer to an ‘unexplained cessation of eruption’ or a ‘poorly understood condition’ [7–9], with observed clinical signs more representative of PFE. In cases with a high likelihood of PFE, it is useful to look for variants of the gene *PTH1R.* While genetic testing is not accessible for some, determining whether there is a family history is a powerful alternative.

In case of ankylosis, a key differential diagnosis, the dental root surface fuses with the surrounding bone.The pathogenesis of ankylosis is less known but has been tied to a history of trauma [10, 11] reported that the presence of the epithelial cell rests of Malassez are essential in preventing the fusion between the alveolar bone and the tooth. Overexpression of WNT is responsible for cementum overgrowth and ankylosis [12]. Irrespective of this distinguishing histologic characteristic, it is difficult and often near impossible to definitively diagnose ankylosis. Practically speaking the diagnosis of ankylosis is based more on ruling out other causes of eruption failure and on the clinical context (i.e. usually isolated).

Failure of eruption of permanent molars is the rarest impaction of permanent teeth [13]. This finding is always challenging for the dentist, even more so when it affects a child. From a clinical point of view, it is very difficult to distinguish between PFE and ankylosis during an early diagnostic process. In both cases, the affected teeth fail to erupt or occupy an infra-occluded position. The ankylosed teeth should elicit a high metallic sound on percussion [14] and have reduced or absent mobility [15]. However, relying on differences in percussive sounds elicited upon tapping with an instrument can vary greatly by operator and it may not be reproducible. In contrast, deploying an Osstell Mentor (Osstell, Gothenburg, Sweden) that detects resonance frequency analysis, as used in implantology, offers some promise [16]. In case of PFE, affected teeth are normally mobile, but become ankylosed in response to an orthodontic force. In case of suspected ankylosis, an orthopanoramic radiographic examination does not clearly and definitively reveal the absence of the periodontal ligament, especially in multi-rooted teeth. As Raghoebar et al. demonstrated [17, 18], the areas of ankylosis are small and often located between the roots.

The early diagnosis is based on the clinical observation of a delay in first molar eruption, and on the signs detected on orthopanoramic x-ray of dental arches. Unfortunately, the differential diagnosis is very difficult. During childhood, it is easier to identify the causes of Mechanical Failure of Eruption (MFE), mostly due to the lack of space in the arch or the presence of cysts or supernumerary teeth that prevent eruption. Another possible cause of the lack of presence of the first permanent molars in the arch is the eruption delay, which alters the timing of the development of the occlusion, but not the eruption mechanism [19].

Since 2015, our research group has carefully curated a collection of clinical cases with eruption failure, and carried out genetic testing for *PTH1R* variants. Our observation and characterization of a large series of cases with failure of eruption of first permanent molars includes determination of the genotype-phenotype correlation and identifying clinical differences between ankylosis and PFE.

In this study, we highlighted the clinical differences and signs that can help establish an early differential diagnosis between PFE, ankylosis, and MFE.

## Methods

### Patients

A cohort of 34 patients aged between 7 and 12 years (mean ± SD: 9.3 ± 1.28 years) with anomalies in the eruption of the first permanent molars was studied in the period from December 2015 to January 2024. 30 patients were part of a new cohort recruited in the period from December 2020 to January 2024 in a multicentric study.

The diagnosis of first molar eruption failure was made on orthopanoramic radiographs and intra-oral photographs of the patients. It was generally defined as failure in primary eruption without any of the following conditions: other impacted teeth, bony or radio-opaque lesions, extensive caries, deciduous caries or crown restorations, or a history of facial trauma. Furthermore, genetic testing was performed to search for variants of the *PTH1R* gene and variants in 27 patients who presented the typical signs of PFE or were highly doubtful. The possible familiar nature of the condition was also investigated on the first-degree relatives.

The study protocol was written in accordance with the Declaration of Helsinki and it was approved by the Ethics Committee of the Università Cattolica del Sacro Cuore, Roma (ID 565-11/2015). Patients were recruited by analyzing the iconographic data (orthopanoramic radiographs and clinical images), collected by orthodontists asking for the genetic test for *PTH1R* variants to confirm the suspicion of PFE. The inclusion criteria were the following: at least one permanent molar with incomplete eruption (infraoccluded), suspected PFE, but uncertainty in diagnosis. Clinical data were ranged following the indications of the clinical findings published in Grippaudo et al. [9].

The dental developmental stage was assessed according to the Nolla index [20] to compare the involved tooth with the contralateral one. Two experienced orthodontists evaluated the clinical records independently, and if they disagreed, they enlisted a third orthodontist to examine the case.

### *PTH1R* sequencing analysis

DNA samples were collected using a cytobrush (Cooper Surgical, Trumbull, CT, USA) and extracted with the QIAamp DNA mini kit (code 51304, Qiagen). Amplifications and nucleotide sequencing of intronic/exonic regions of *PTH1R* gene (NM_000316.3) were carried out as described in Grippaudo et al. [9]. All sequences were aligned to the reference genome (GRCh38/hg38) and the frequency of variants in the general population was checked on the Genome Aggregation Database (GnomAD) (https://gnomad.broadinstitute.org/) and the Single Nucleotide Polymorphism Database (dbSNP) (https://www.ncbi.nlm.nih.gov/snp/). The potential pathogenicity of *PTH1R* variants was searched in the NCBI ClinVar database (https://www.ncbi.nlm.nih.gov/clinvar/).

### Statistical analysis

A descriptive statistical analysis was completed to analyze the data. The frequency of key clinical traits in PFE, ankylosis and MFE was analyzed using GraphPad Prism v.8 software (San Diego, CA, USA). Specifically, Fisher’s exact test was employed to evaluate the significant association between variables (clinical traits as reported in Table 1), i.e., group with PFE, group with ankylosis and group with MFE. The significance level was set at *p* ≤ 0.05.

## Results

Despite the rarity of the condition, the modest number of recruited patients still allows our assessment to draw useful information for early differential diagnosis.

It is very difficult to distinguish PFE from ankylosis at an early stage, especially when only one first molar is involved. Likewise, identification of MFE can be difficult in the absence of a clear understanding of the natural history of an affected patient’s dentition.

**Fig. 1:**
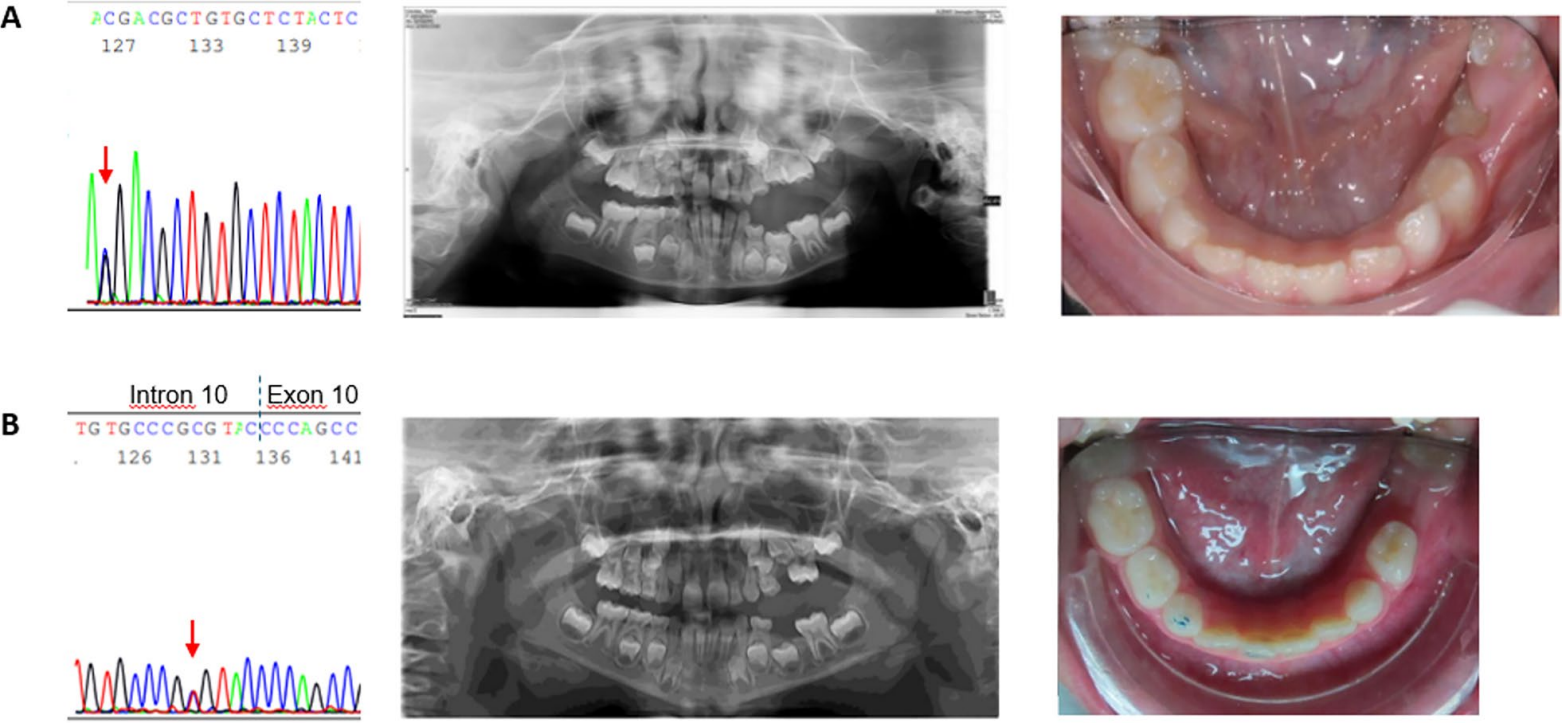
Two *PFE cases and corresponding variants in PTH1R gene.***A** Case 1 presents the variant in exon 9 of the *PTH1R* gene, c.720 G> C;p.(Lys240Asn) (left panel). Note the teeth anomalies in the OPT (middle panel) and in the intra-oral photograph (right panel). **B** In case 3 the c.988+5G> A (rs779366145) variant in intron 10 is reported (left panel). OPT of the child (middle panel) and intra-oral photograph (right panel) show the teeth anomalies (i.e. first molar retention). Both sequences of the *PTH1R* gene are reported in the reverse strand

Out of 34 cases, we found only 3 patients with PFE confirmed by the presence of *PTH1R* variants (Fig. 1). This finding further confirms the rarity of PFE. In one PFE case with the most severe phenotype, there was a bilateral presentation (case #2), previously reported [9]. In the 2 other cases the eruption pathway was more obviously clear, and presented as a less severe infraocclusion. It is important to report that in all three cases of PFE, one of the two parents showed clinical signs of PFE and presented the same *PTH1R* variant of her/his son/daughter, confirming the importance of familiarity.

In our cohort of patients, 12 presented with clinical signs suggestive of MFE diagnosis. Three cases (#27, #28 and #31) had eruption failure of all molars. In these cases, the role of a mechanical obstruction was very probable or evident due to the presence of cysts (#42, #54), space deficit (#25, #54, #34), agenesis and infraoccluded deciduous second molars (# 24, #28, #29, #31, #32, #33), tongue pressure (#28, #29, #30, #31), delayed eruption (#33).

The remaining 19 cases without variants of the *PTH1R* gene were considered cases of ankylosis. Confirmation of an ankylosis diagnosis can be increased if extraction of the putative ankylosed tooth results in normal orthodontic movement of the remaining teeth [21].

The clinical results are presented according to the observation of the main clinical signs reported in Table 1.

**Table 1:**
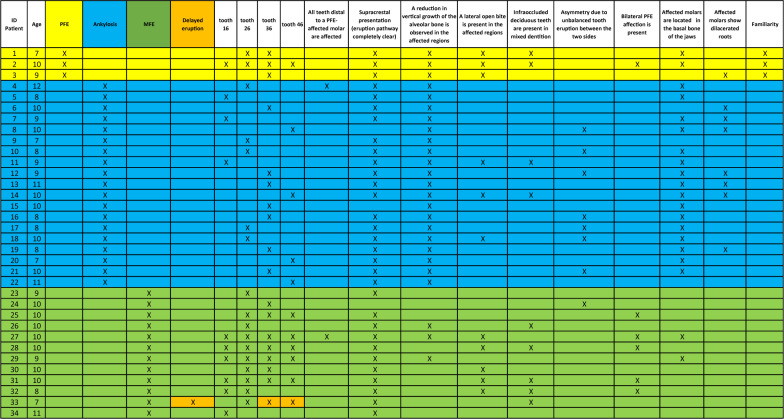
Characteristics of the patient cohort examined

### Teeth distal to a PFE-affected molar are also affected

In such an early stage the second molars were in the bone, in a developmental stage. So, it is difficult to judge if they will be affected in the future. For the same reason, we cannot assess if PFE cases had a Type I or Type II presentation.

### Supracrestal presentation (eruption pathway completely clear)

The clinical sign that leads us to suspect the arrest of the tooth’s eruptive process is the resorption of the bone along the eruption path. Only in two cases, diagnosed as ankylosis (#8, #15), the eruptive path is not clear. In case #8 the bone is not resorbed. During follow-up, an eruptive cyst forms which partially resorbs the bone. In case #15, a cyst has been observed around the crown of the tooth since the first x-ray in 2022 and in the follow-up in 2023. These two cases were classified as ankylosis because the affected teeth showed roots close to the basal bone as if the eruption process had never started.

Case #8 presents an ankylosed molar that developed a cyst during follow-up. This observation highlights how a late diagnosis can present itself with a different appearance, generating diagnostic ambiguity.

**Fig. 2:**
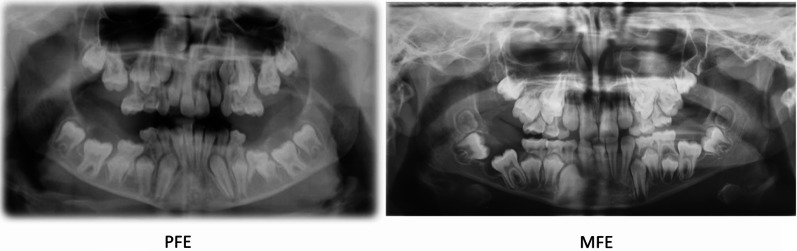
Comparison between patient #2 (PFE) and #27 (MFE) with similarity of clinical signs and different genetic test result

The presence of odontogenic cysts may cause a mechanical obstruction to dental eruption, but is not always necessary and sufficient to render a diagnosis of MFE. In our sample, we observed agenesis of the adjacent second premolar coexists (patient #24), apparently without any correlation with infraocclusion of the permanent first molar. Patients #2 and #27 present many similarities (Fig. 2): the first molars are all unerupted, and the lower molars have cysts around the crowns. In patient #2 a diagnosis of PFE is confirmed, while in patient #27 a diagnosis of MFE is confirmed. In patient #27 we didn’t find pathogenetic variants. The patient’ analysis reported two benign variants, in heterozygous the intronic c.1116 + 58 T > C (rs1531137) variant and the synonymous c.1389 T > C;p.(Asn463=) (rs1138518) variant confirmed the clinical suspicion of MFE diagnosis. The upper first molars could be blocked by the second deciduous molars, in a situation of lack of space in the dental arch. In the panoramic radiograph, a radiolucent space between lower first molars and the surrounding bone is observed which indicates the presence of the periodontal ligament, supporting the hypothesis of MFE.

### There is a reduction in vertical growth of the alveolar bone in the affected regions - 

#### Affected molars are located in the basal bone of the jaws -

##### Affected molars show dilacerated roots

**Fig. 3:**
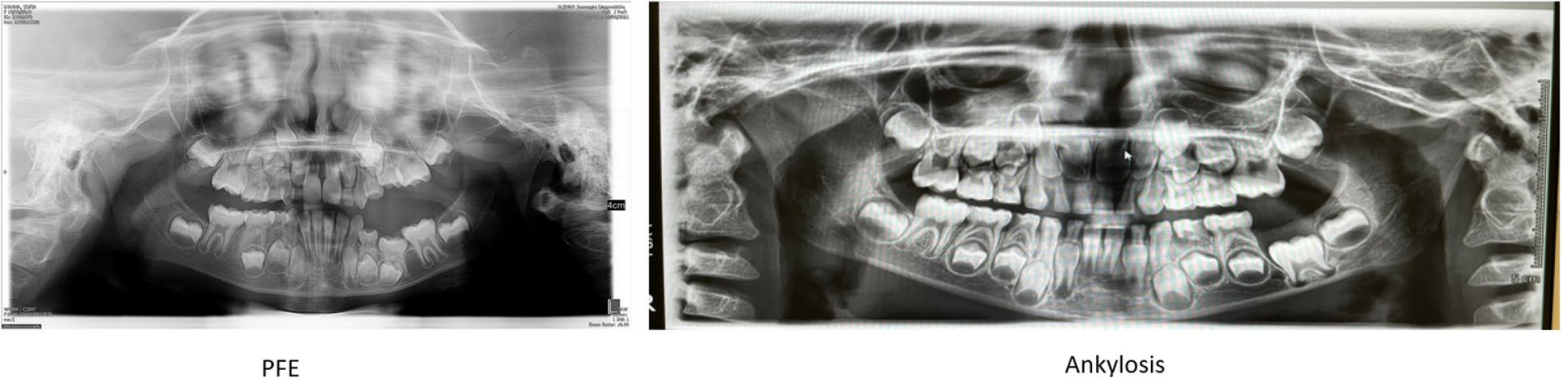
Patient #1 (PFE) and patient #12 (Ankylosis): in both a reduction in the vertical growth of the alveolar bone is observed, which is greater in the case of Ankylosis

Roots in ankylosed teeth were located in the basal bone and often dilacerated. The OPT did not always allow observation of the PDL status (i.e., completely intact). The reduction of vertical growth of the alveolar bone is present in both PFE and in ankylosis cases (Fig. 3). However, in PFE, affected mandibular teeth show a distance from the lower edge of the jaw, while ankylosis teeth are almost always in contact with the lower edge of the jaw.

##### A lateral open bite is present in the affected regions

Bilateral cases were rare in ankylosis cases, and open bite was present in three cases (patients #11, #14, #18). In patient #18 it become more evident in the follow up, due to the inappropriate orthodontic therapy.

##### Deciduous infraoccluded in mixed dentition

**Fig. 4:**
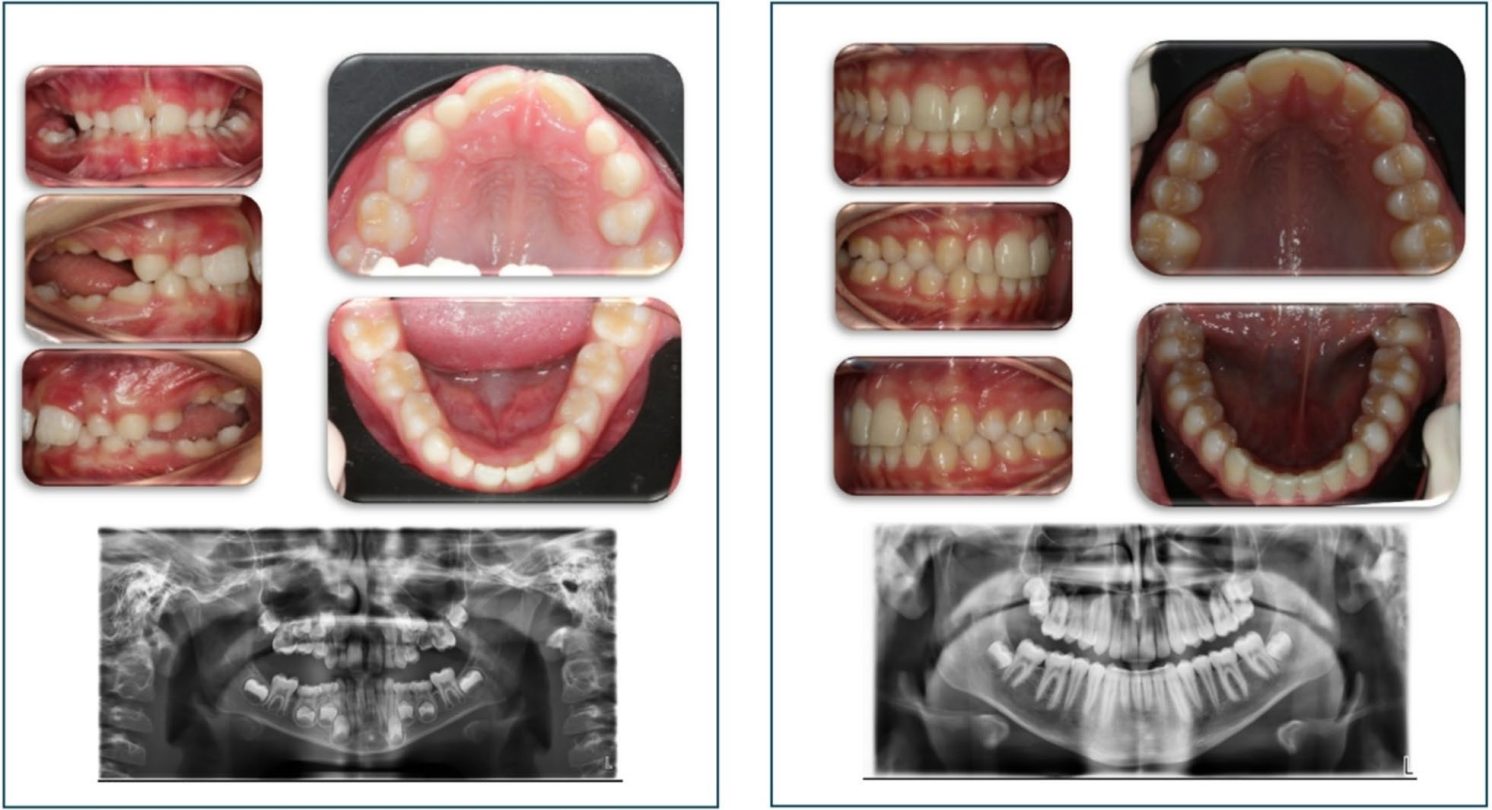
Patient #31: **A** at the time of diagnosis; **B** after orthodontic therapy

Deciduous molars were infraoccluded in two out of three cases of PFE. Interestingly, in four cases ankylosis of deciduous teeth caused MFE. In one case (#31), who underwent orthodontic therapy, the clinical result was excellent, with a complete recovery of the molar eruption (Fig. 4).

##### Asymmetry due to bilaterally unbalanced eruption of the teeth

Six cases of ankylosis were characterized by asymmetry due to bilaterally unbalanced tooth eruption. According to the Nolla classification, the tooth with ankylosis was one step earlier in the developmental stage than the contralateral one. This sign was absent in cases of MFE and PFE.

##### Affected individual presents with a bilateral affection of PFE

**Fig. 5:**
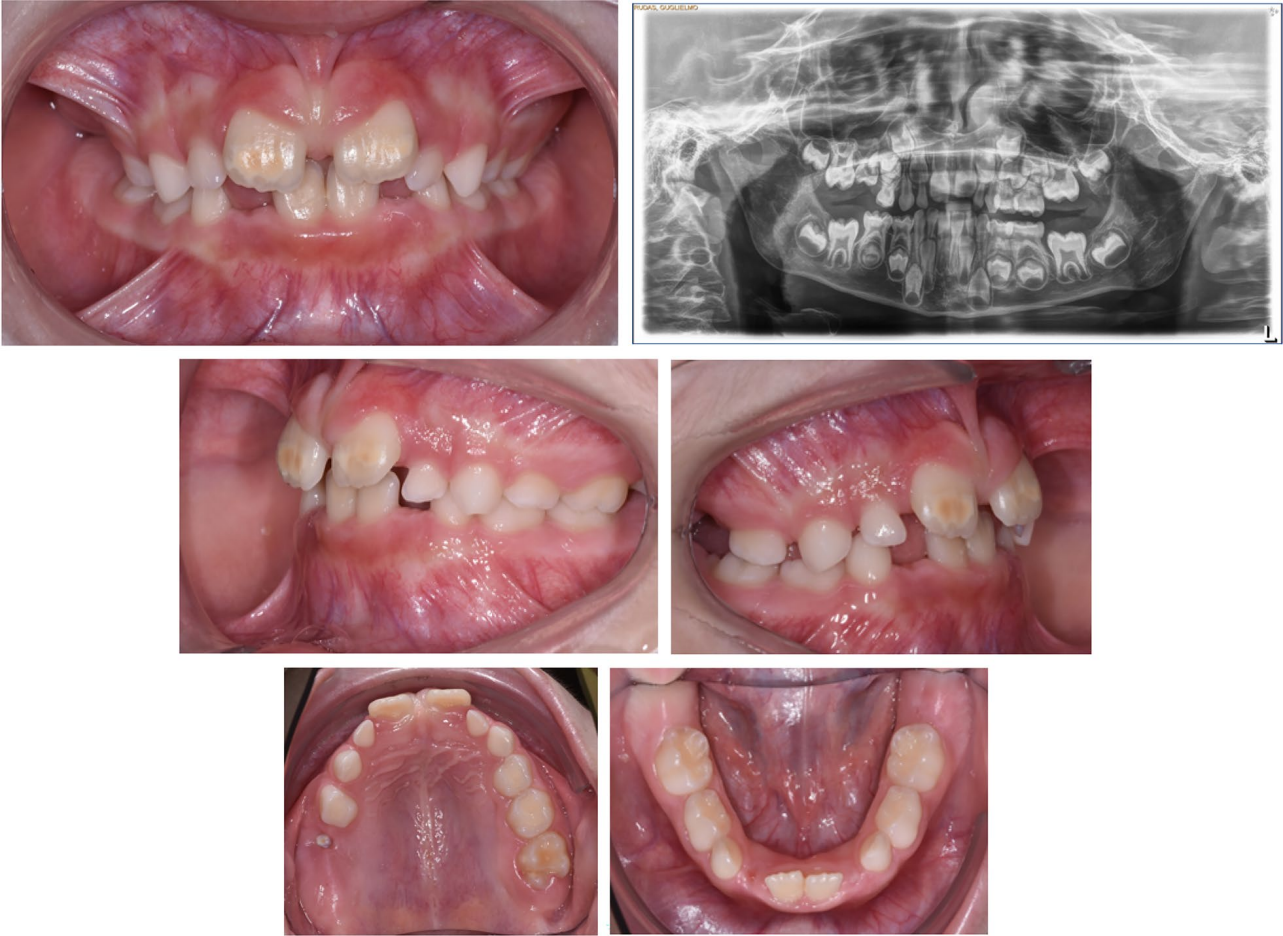
Patient #33 presenting infraocclusion of the element 55 and agenesis of the element 15 with delayed eruption of 36 and 46

Bilateral affection was observed in seven patients (20,5%). One patient had a severe PFE (#2). The remaining patients had MFE: two of them showed a tongue thrust, with bilateral open bite, while in one case (#33) it was difficult to understand the cause underpinning the clinical sign. We posit that MFE in this scenario is caused by the ankylosis of the second upper deciduous molar for the upper right first molar, and a delay in eruption of the lower first molars, but it will be necessary to follow up the patient to confirm the diagnosis (Fig. 5).

In our cohort, we found that the most involved teeth were the lower and upper left first molars, but this observation may not represent statistical or scientific significance.

### PTH1R sequencing analysis

Out of 34 patients, we performed *PTH1R* sequencing analysis in 27 cases. We found 20 patients with variants in *PTH1R*: 3 with PFE diagnosis, 12 with ankylosis, and 5 with MFE diagnosis (Table 2).

**Table 2:**
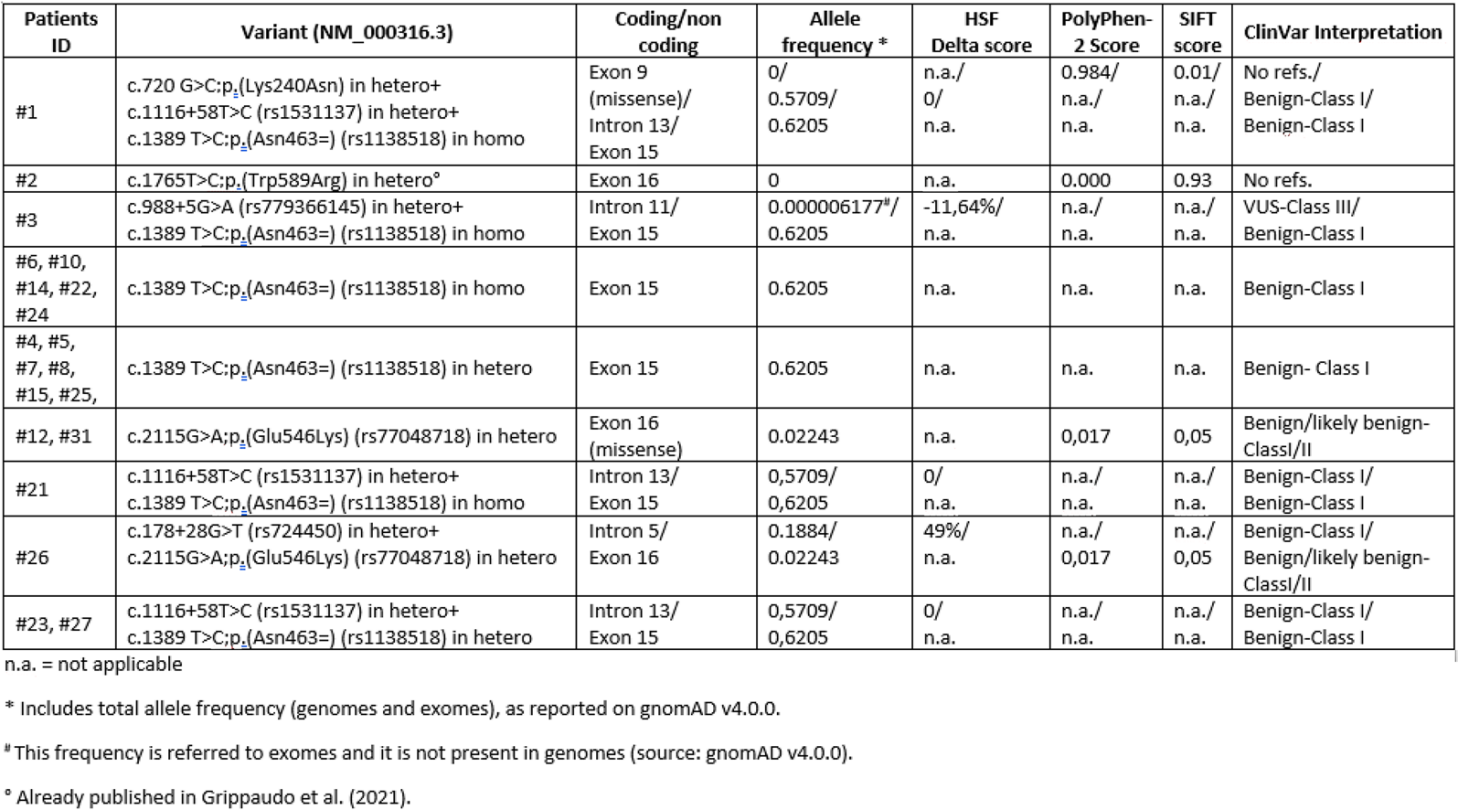
List of the variants reported in our cohort of patients

Allele frequency and prediction tools analysis are shown

In addition to benign variants, the three PFE cases presented the following variants: in patient #1 the missense c.720 G > C;p.(Lys240Asn) variant in heterozygous (in exon 9); in patient #2 the missense c.1765T > C;p.(Trp589Arg) variant in heterozygous (exon 16); in patient #3 the c.988 + 5G > A (rs779366145) in heterozygous (in intron 11). The missense variant found in patient #1 was never reported in gnomAD and ClinVar databases. Based on two different prediction tools (PolyPhen-2 and SIFT), the possible impact of the p.(Lys240Asn) amino acid substitution resulted negative on the function/structure of the receptor. This variant was inherited from her mother (not included in this cohort of patients). The missense variant found in patient #2 was previously reported and discussed in Grippaudo et al. [9]. The intronic variant found in patient #3 has been classified as of uncertain significance (VUS). It was never reported in ClinVar and with a very low frequency in gnomAD database. Evaluating the impact of this variant with HSF (Human Splicing Finder) tool gave back a delta score of −11,64%, indirectly supporting its pathogenicity. Patient #3 inherited the variant from his father (not included in this cohort of patients). Because of rarity and correlation with the clinical findings, these three variants were considered associated with the phenotype. The most frequent benign variant found in 15 of our cohort of patients was the exonic synonymous c.1389 T > C;p.(Asn463=) (rs1138518) variant reported in homozygous in two PFE cases (#1 and #3), four patients (#6, #10, #14 and #22) with ankylosis and one (#24) with MFE diagnosis, and in heterozygous in six patients (#4, #5,#7, #8 and #15) with ankylosis and three (#23, #25 and #27) with MFE.

### Statistical results

Based on data reported in Table 1, the supracrestal presentation (with an eruption pathway completely clear) and the asymmetry due to bilaterally unbalanced eruption of the teeth did not represent key clinical signs that may help in the differential diagnosis of the three conditions (PFE, ankylosis and MFE: no statistical significance in the comparisons between groups). In the comparison between PFE and ankylosis groups, the presence of lateral open bite (in the affected region) (*p* = 0.0011), of infraoccluded deciduous teeth in the mixed dentition (*p* = 0.0179) and asymmetry due to bilaterally unbalanced eruption of the teeth (*p* = 0.0081) represented distinctive clinical signs reaching statistical significance compared to the other clinical findings. Between PFE and MFE, the distinctive clinical sign that reached statistical significance was the reduction of vertical growth of the alveolar bone in the affected regions (*p* = 0.0152). MFE may be distinguished from ankylosis by the following clinical findings: the reduction of vertical growth of the alveolar bone in the affected regions (*p* < 0.0001), presence of infraoccluded deciduous teeth in the mixed dentition (*p* = 0.0449), bilateral affection of teeth (*p* = 0.0013), affected molars located in the basal bone of the jaws (*p* < 0.0001) and affected molars with dilacerated roots (*p* = 0.0160).

## Discussion

Early diagnosis of dental eruption failure is crucial to adopt the correct therapeutic measures and prevent the clinical situation from becoming more complex with growth. In the case of MFE, removing obstacles to eruption during the completion of root formation favors the prognosis of recovery of the correct position of the molars. With regard to ankylosis, it is demonstrated that during adolescence signs of facial asymmetry appear [22]. In the case of PFE, early diagnosis serves to avoid unnecessary orthodontic therapies.The observation of clinical signs in children with eruption problems of the first permanent molars has led us to formulate hypotheses that can aid in an accurate diagnosis, distinguishing PFE from ankylosis and MFE at an early presentation. In PFE the tooth begins to erupt, while in ankylosis it remains closer to the basal bone: ankylosis prevents the tooth from erupting, while the tooth affected by PFE has an absent or reduced capacity to erupt depending on the incomplete penetrance of the *PTH1R* gene [23–27].

If the tooth partially erupts or remains impacted and has roots in the alveolar bone, but is not PFE, the causes of MFE must be sought. The observation of the distance from the basal bone in the maxilla is difficult to evaluate by looking at orthopanoramic radiographs. In case of doubt, an evaluation on cone beam computed tomography (CBCT) could be useful.

Cases with ankylosis demonstrate a more basal position than those with PFE. One hypothesis of explanation could be related to the genetic defect at the origin. If the problem is the absence of Malassez rests [11] or aberrantly elevated Wnt signaling, root development is compromised from the onset of formation. In the case of PFE, *PTH1R* haploinsufficiency can manifest itself in different phenotypic forms, and the involved teeth begin eruption and then stop it [26, 28, 29].

Infraoccluded primary teeth represent a clinical sign related to PFE [27]. This finding is confirmed in two out of three of our cases with PFE described here. However, we also found this clinical sign in five cases with MFE, where no variants of the *PTH1R* gene were found. When infraocclusion of deciduous teeth is associated with a class III developmental pattern and a lateral interposition of the tongue [30], the lateral open bite improves during time. Tieu et al. [31] reported that, in case the ankylosis of the second deciduous molars is an obstacle to the eruption of the adjacent first permanent molars, the extraction is needed [31–33]. The observation of the patient in permanent dentition, after the removal of the deciduous in ankylosis, does not allow us to understand the dynamics of the arrest of eruption of the permanent molar.

In the group of patients examined, the familiarity of the pathology was observed only in subjects with PFE. This is further proof of the importance of the family pathogenetic history.

Table 3 highlights the main clinical signs that can help in early differential diagnosis. Unfortunately, they are not sufficient to identify the problem, and in case of doubt, a more in-depth diagnostic investigation including genetic testing and periodic observation is suggested.

**Table 3:** Showing the main clinical signs to consider in order to orient oneself in the differential diagnosis. In case of doubt, genetic testing and periodic observation of the natural evolution are recommended

PFE	Ankylosis	MFE
Mono-lateral open bite or more evident on one side	Tooth development slower than the contra-lateral tooth	Bilateral open bite
Deciduous teeth infra-occluded	Dilacerated root	Deciduous teeth infra-occluded that obstruct the eruption path of the permanent molars and Ankylosis
Familiarity	Root in the basal bone	Crowding in the posterior area
Reduction in vertical bone growth	One side affected, can also affect growth	Tongue thrust or large tongue

### Limitations

The diagnosis of ankylosis should be confirmed using 3D radiographs. However, this test, which involves the provision of a higher dose of X-rays to a child, must be limited to the most doubtful cases and in which it is intended to proceed with the recovery of the tooth in the arch.

## Conclusions

The failure of molar eruption is a rare event, which can occur accompanied by some clinical signs that can help formulate a differential diagnosis between PFE, MFE and ankylosis. However, during development and tooth exfoliation, in the presence of impacted or partially erupted permanent molars, the clinical appearance can become complicated. It then becomes more difficult to distinguish between these pathologies, which have a different prognosis and require specific treatment plans. In adult patients, the diagnosis in case of doubt may be based on the result of genetic testing for *PTH1R* gene variants, but it may be difficult to plan an effective treatment plan.

Early diagnosis can help avoid complications due to alteration of dental occlusion. Furthermore, it may be easier to detect diagnostic differences, sometimes even without resorting to genetic testing.

The fact remains that, in the most doubtful cases, it is advisable to carry out the genetic test, because in this field there are still many points that require clarification and progress in the knowledge provided by scientific research.

## Data Availability

No datasets were generated or analysed during the current study.

## Abbreviations

PDL: Periodontal ligament
PFE: Primary failure of eruption
MFE: Mechanical failure of eruption
PTHLH: Parathyroid hormone-related protein
*PTH1R*: Parathyroid Hormone 1 receptor
CBCT: Cone beam computed tomography
